# Ancient human miRNAs are more likely to have broad functions and disease associations than young miRNAs

**DOI:** 10.1186/s12864-017-4073-z

**Published:** 2017-08-31

**Authors:** Vir D. Patel, John A. Capra

**Affiliations:** 1 Department of Biology, Duke University, Durham, NC 27708 USA; 2 Department of Biology, Western Kentucky University, Bowling Green, KY 42101 USA; 3 Departments of Biological Sciences, Biomedical Informatics, and Computer Science, Vanderbilt Genetics Institute, Center for Structural Biology, Vanderbilt University, Nashville, TN 37232 USA

**Keywords:** miRNA evolution, Human disease, Phylogenetics, Gene regulation

## Abstract

**Background:**

microRNAs (miRNAs) are essential to the regulation of gene expression in eukaryotes, and improper expression of miRNAs contributes to hundreds of diseases. Despite the essential functions of miRNAs, the evolutionary dynamics of how they are integrated into existing gene regulatory and functional networks is not well understood. Knowledge of the origin and evolutionary history a gene has proven informative about its functions and disease associations; we hypothesize that incorporating the evolutionary origins of miRNAs into analyses will help resolve differences in their functional dynamics and how they influence disease.

**Results:**

We computed the phylogenetic age of miRNAs across 146 species and quantified the relationship between human miRNA age and several functional attributes. Older miRNAs are significantly more likely to be associated with disease than younger miRNAs, and the number of associated diseases increases with age. As has been observed for genes, the miRNAs associated with different diseases have different age profiles. For example, human miRNAs implicated in cancer are enriched for origins near the dawn of animal multicellularity. Consistent with the increasing contribution of miRNAs to disease with age, older miRNAs target more genes than younger miRNAs, and older miRNAs are expressed in significantly more tissues. Furthermore, miRNAs of all ages exhibit a strong preference to target older genes; 93% of validated miRNA gene targets were in existence at the origin of the targeting miRNA. Finally, we find that human miRNAs in evolutionarily related families are more similar in their targets and expression profiles than unrelated miRNAs.

**Conclusions:**

Considering the evolutionary origin and history of a miRNA provides useful context for the analysis of its function. Consistent with recent work in Drosophila, our results support a model in which miRNAs increase their expression and functional regulatory interactions over evolutionary time, and thus older miRNAs have increased potential to cause disease. We anticipate that these patterns hold across mammalian species; however, comprehensively evaluating them will require refining miRNA annotations across species and collecting functional data in non-human systems.

**Electronic supplementary material:**

The online version of this article (doi:10.1186/s12864-017-4073-z) contains supplementary material, which is available to authorized users.

## Background

MicroRNAs (miRNAs) are small, non-coding RNAs found in eukaryotic cells that post-transcriptionally regulate mRNA targets [1]. miRNAs are fundamental elements of eukaryotic genetic regulatory networks [2]; they have been implicated in many cellular developmental processes including proliferation, apoptosis, and mitotic progression [3–5]. Excessive up-regulation or down-regulation of miRNAs along with aberrations in both target and miRNA nucleotide sequences can induce disease [6]. For example, over- and under-expression of miRNAs can disrupt cellular differentiation in patterns characteristic of cancer cells [7]. In total, hundreds of miRNAs have been associated with human disease [8, 9].

miRNAs can be divided into evolutionarily related families derived from common ancestral sequences. miRNAs in these families often play important biological roles through redundant or concerted pathways [10–15]. Knowledge of the evolutionary history of a protein-coding gene often provides insight into the function of the protein it encodes [16, 17]. For instance, genes responsible for fundamental cellular processes are often as old as the last common ancestor of all life, while genes involved in cellular communication, a trait associated with multicellular organisms, are enriched for origins at the dawn of animal multicellularity.

Several studies have revealed that the evolutionary dynamics of miRNA birth and change of function are significantly faster than for protein-coding genes [18, 19]. However, the extent to which evolutionary context is informative about miRNA function, expression, and targets remains unresolved [19–22]. For example, recent studies have come to differing conclusions on whether miRNAs gain or lose target genes and expression in more tissues over evolutionary time as they are integrated into functional networks of the cell [18, 19, 21, 22]. Our understanding of the evolutionary relationships between human miRNAs, their gene targets, their expression, and their influence on disease remains incomplete.

In this study, we comprehensively characterized the connections between miRNA evolutionary origins, functional dynamics, and relationship to human disease. We found that the evolutionary age of a miRNA is strongly correlated with its likelihood of contributing to disease. Furthermore, as was observed for protein-coding genes, human diseases vary in the evolutionary origins of their associated miRNAs. Consistent with the increased disease associations of miRNAs with age (and recent results in Drosophila [21]), we observed an increasing regulatory influence of human miRNAs with age. Older miRNAs have greater breadth of expression across tissues and target more genes than younger miRNAs. Altogether, our analyses reveal consistent relationships between miRNA evolution, function, and disease.

## Results

### The phylogenetic age distribution of human miRNAs

**Fig. 1:**
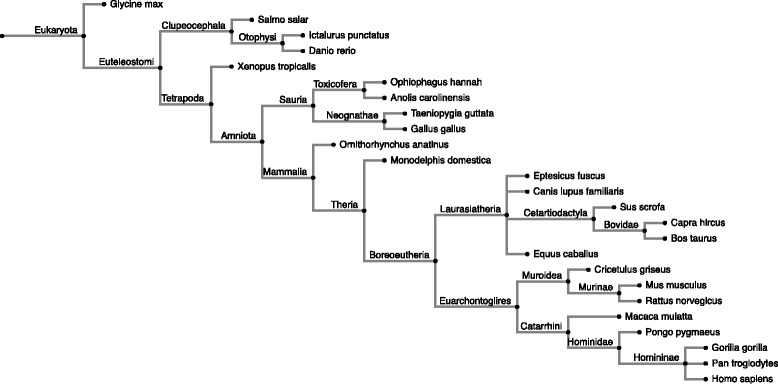
Phylogenetic tree for 25 of the species analyzed. This tree represents the phylogenetic relationships between the 25 species with the most miRNA annotations in the miRBase dataset. The tree was built from the NCBI taxonomy database using the interactive tree of life (ITOL) server. Branch lengths are not to scale. The full list of 146 species considered in this study is available in Additional file 1

**Fig. 2:**
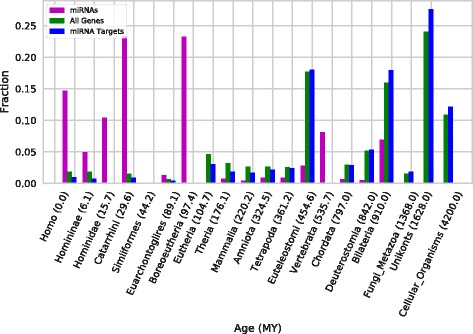
Human miRNAs are younger than protein coding genes. This figure compares the age distribution of human miRNAs (*purple*) and genes (*green*). The evolutionary origins of human miRNA are significantly younger than genes (*P* = 2.5e–300, Mann-Whitney *U* test). Human miRNA are also significantly younger than the genes they target (*blue*). Note that PC genes were not assigned to several branches (Hominidae, Simiiformes, Boreoeutheria, and Vertebrata) due to fewer species being present in the gene dataset. The lower resolution for gene ages does not influence our conclusions, because the genes are assigned the lower bounds on their ages

We computed the phylogenetic age of 1025 human miRNAs from miRBase by applying a modified version of ProteinHistorian [23] to all annotated miRNAs from a set of 146 species (Fig. 1; Additional files 1 and 2). We found that, consistent with previous work, the majority of human miRNAs are young: 46% are primate-specific and 14% are human specific (Fig. 2). The most common origins of miRNAs that have survived to the present are on the branches leading to the last common ancestors of all Boreoeutheria—about 100 million years (MY) ago—and Old World monkeys and apes (Catarrhini; ~20 MY ago). Our data also suggest that the miRNAs originating on the branches leading to Coelomata and Vertebrata comprise most of the ancient human miRNAs.

To evaluate whether the timing and dynamics of human miRNA emergence mirrored that of protein-coding genes, we compared the distribution of miRNA ages to protein ages computed over a similar set of species. Compared to miRNA ages, the protein age distribution is significantly shifted toward ancient origins (Fig. 2; *P* = 2.5e–300, Mann-Whitney U test). The average age of human miRNAs in our dataset is 169.9 MY while the average age for human proteins is 1195.1 MY. This is not surprising given that proteins could have origins in the last common ancestor of all life while miRNAs are a more recent evolutionary innovation. Nonetheless, the miRNAs are dramatically more likely to have recent origins.

### Older miRNAs are associated with more diseases than younger miRNAs

**Fig. 3:**
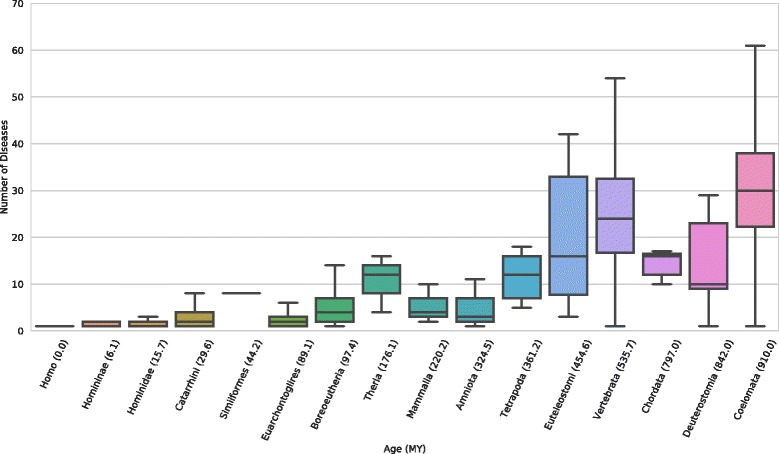
The number of diseases associated with a miRNA significantly increases with its age. Each *box* and *whisker plot* gives the median, upper and lower quartiles, and 1.5 times the inner-quartile range of the number of diseases associated with miRNAs of each age. The Spearman’s correlation between miRNA age and the number of diseases is 0.78 (*P* = 7.4e–103)

Using 383 miRNA–disease associations taken from the Human microRNA Disease Database (HMDD) [8], we explored the relationships between the age of a human miRNA and its disease associations. Overall, 52% of human miRNAs are associated with at least one disease. The age of a miRNA is significantly positively correlated with its number of disease associations (Fig. 3; Spearman’s ρ = 0.78; *P* = 7.4e–103). On average, young miRNAs are associated with very few (<10) diseases, while the oldest miRNA are associated with many (~30) diseases. These results include all miRNAs, but the trends were similar when only disease-associated miRNAs were considered.

The HMDD database contains associations with many similar diseases, so it is possible that the more fine-grained coverage of certain disease classes, like cancer, might bias the number of diseases associated with certain miRNAs. To account for this, we collapsed cancers within HMDD and found that miRNA age and number of disease associations are still significantly correlated (Additional file 3). To further control for relatedness between diseases, we mapped each of the HMDD disease terms to the hierarchical Medical Subject Headings (MeSH) database. This enabled us to collapse diseases using MeSH’s hierarchy of disease classes and to compare miRNA-disease relationships at differing levels of specificity (Additional file 3). At the most general level of MeSH disease classification, we found an even stronger relationship between miRNA age and number of disease associations (Spearman’s ρ = 0.77; *P* = 2.7e–108).

### Diseases differ in the age of their associated miRNAs

**Fig. 4:**
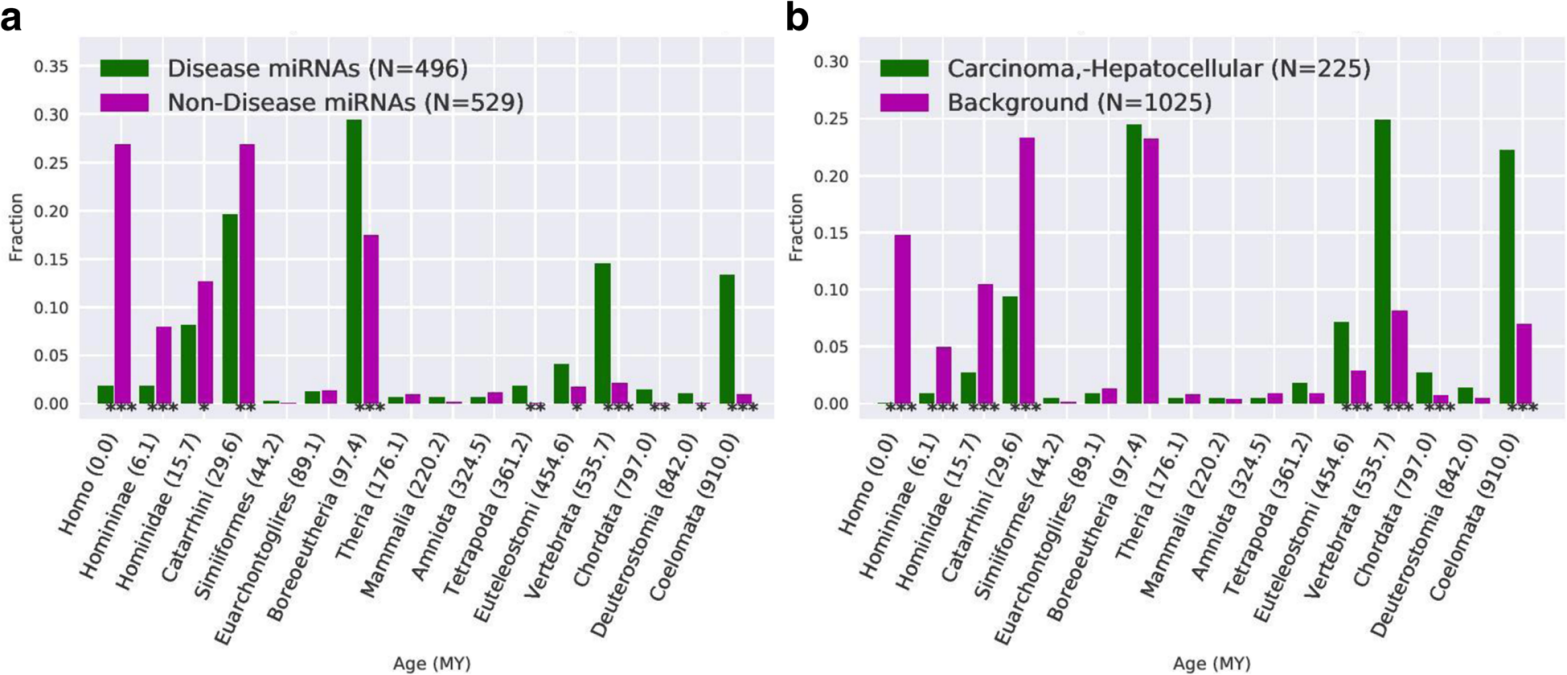
miRNAs associated with disease are older than expected from the background miRNA age distribution. (**a**) The median age of the miRNAs associated with disease is 97.4 MY compared to 26.9 MY for non-disease miRNAs. This relationship also holds at the individual disease level (Additional file 3). (**b**) The median age of the miRNAs associated with hepatocellular carcinoma is 535.7 MY; this is significantly older than expected if they were randomly drawn from all miRNAs (*P* = 2.5e–38, Mann-Whitney *U* test). Cancers are particularly enriched for old miRNA; the miRNAs associated with 80% of all cancers in our dataset are significantly (*P* < 0.01) older than expected by chance. Asterisks indicate a significant difference in the number of miRNA associated with disease of a given age than expected (“***” for *P* < 0.001, “**” for *P* < 0.01, and “*” for *P* < 0.05)

The genes associated with different human diseases often have distinct evolutionary origin profiles; for example, genes associated with cancer are enriched for origins near the development of animal multicellularity [17, 24]. Motivated by this association between gene age and disease, we compared the age distributions of miRNAs associated with 383 diseases to the background miRNA age distribution. Diseases differ significantly in the average age of their associated miRNAs (Additional file 3). However, as suggested from the fact that older miRNAs are more likely to be involved in disease than younger miRNAs (Fig. 3), disease-associated miRNAs are significantly older than miRNA not associated with disease (Fig. 4a; median 97.4 vs. 29.6 MY; *P* = 1.9e–60, Mann-Whitney *U* test).

Tested individually, 211 out of 383 diseases, including many cancers, are associated with miRNAs that are significantly older than the background of all miRNAs (*P* < 0.01). Of these “older” diseases, 48% were cancers, comprising 80% of all cancers in our dataset. For example, the miRNAs associated with hepatocellular carcinoma had a median age of 535.7 MY; this is significantly older than expected from the miRNA background (Fig. 4b; *P* = 2.5e–38). Consistent with the enrichment observed in previous studies of the association of gene age with cancer [24], cancer miRNAs are enriched for origins on the branch that contains the ancestor of all animals, as well as several older branches.

### Older miRNAs target more genes than younger miRNAs

To explore the potential causes of increased disease association among older miRNAs, we evaluated the relationship of other functional attributes of miRNAs with their ages. miRNAs regulate the translation of specific target genes in certain tissues and contexts; thus, we quantified the association between the ages of miRNAs and their known targets and contexts of activity.

As expected from the increasing disease association with age, we found a correlation between the age of a human miRNA and its number of validated gene targets from miRTarBase [25] (Spearman’s ρ = 0.29; *P* = 2.7e-16). This suggests that older miRNAs tend to play more functional roles, and consequently, provide more opportunities for disrupted activity to lead to disease. There is also a modest relationship between the pairwise similarities of miRNA target gene profiles and age (Spearman’s ρ = 0.17; *P* ≈ 0).

**Fig. 5:**
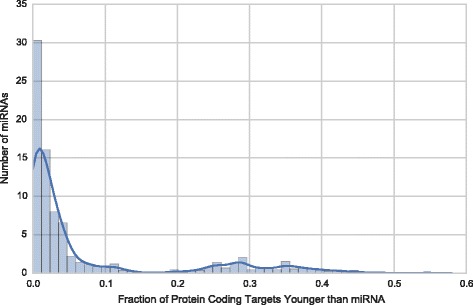
miRNAs are younger than their protein-coding gene targets. This histogram gives the number of human miRNAs (y-axis) for which a given fraction of their gene targets are younger than it (x-axis). On average, 92.5% of a miRNA’s gene targets are older than the miRNA itself; this is significantly more than would be expected from the differences in age distribution between miRNAs and genes (*P* < 0.001, permutation test). In the most common scenario, a miRNA is younger than all of its target genes

The vast majority of miRNAs target genes that are older than they are. miRNAs are younger than 92.5% of their target genes on average (Fig. 5). This is not surprising, given that miRNAs are significantly younger overall than genes (Fig. 2); however, miRNAs are even younger than their target genes than would be expected based on the difference in age distributions (*P* < 0.001, permutation test). Thus, miRNAs mainly target genes that were in existence at the time of their origin. However, beyond this general trend, miRNA ages were only weakly associated with the average age of their targets (Spearman’s ρ = 0.07; *P* = 0.04).

### Older miRNAs are more broadly expressed than younger miRNAs

**Fig. 6:**
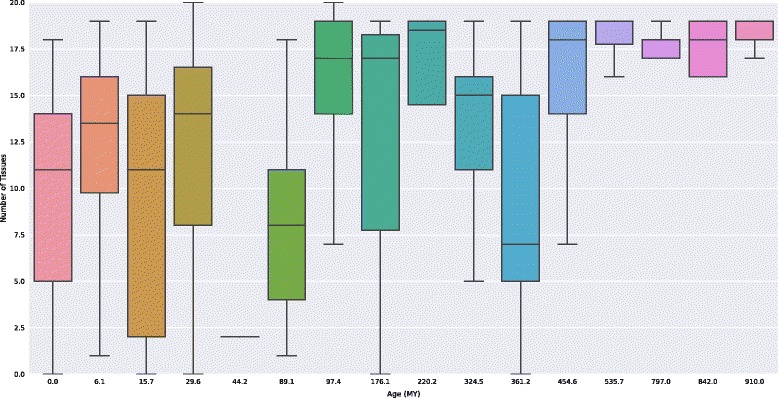
Old miRNAs are expressed in more tissues than young miRNAs. miRNA age is significantly correlated with the number of tissues in which it is expressed (Spearman’s ρ = 0.51, *P* = 8.8e–52). Stratifying miRNAs older and younger than 100 MY revealed that old miRNAs are expressed in significantly more tissues (*P* = 8.8e–27; Mann-Whitney U test). Old miRNAs also show less variation in the number of tissues in which they are expressed

We next examined the relationship between miRNA evolutionary age and tissue expression across 20 human tissues. miRNA age is significantly correlated with the breadth of expression across tissues (Fig. 6; Spearman’s ρ = 0.51, *P* = 8.8e–52). Stratifying old and young miRNAs revealed that old miRNAs are significantly more broadly expressed (*P* = 8.8e–27, Mann-Whitney *U* test). Furthermore, young miRNAs vary widely in their patterns of expression across tissues, while older miRNAs are generally expressed in many tissues (Fig. 6). As a whole, these findings are consistent with the disease association and gene target results in suggesting that older miRNAs have many potentially pleiotropic functions and influence diverse phenotypes, while younger miRNAs are often, but not always, restricted to a subset of tissues.

### Evolutionarily related miRNAs are more similar in their functional dynamics and disease associations

miRNAs can be created from existing miRNAs, e.g., via duplication, or de novo from previously non-coding sequences. The former process has created many “families” of evolutionarily related miRNAs that share common ancestral sequences and are often co-located in the genome [10, 15, 26]. To incorporate this dimension into our analyses, we evaluated our previous results in the context of human miRNA family memberships derived from miRBase.

We first examined the phylogenetic ages of miRNAs in families and found that they differ from those of human “singleton” miRNAs without other family members in the human genome (Additional file 4). The 332 miRNAs in families are generally older than the 693 singletons (89.1 MY vs. 29.6 MY, *P* = 4.6e–5, Mann-Whitney *U* test). However, 68% of miRNA families were primarily composed of young miRNAs. To test whether these differences in evolutionary conservation held at the sequence level as well, we compared PhastCons vertebrate evolutionary conservation scores for both sets of miRNAs. Family miRNAs are also more conserved at the sequence level than singletons, with an average PhastCons score of 514.0 compared to 473.4 (*P* = 2.5e–4, Mann-Whitney *U* test). There was no relationship, however, between the sizes of miRNA family clusters and their levels of conservation.

**Fig. 7:**
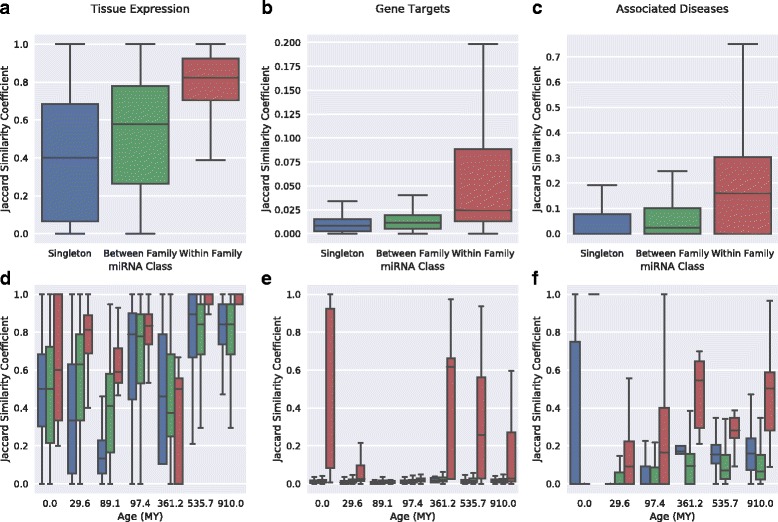
Human miRNAs within the same family are more functionally similar than other miRNAs. (**a**) miRNAs within the same family have more similar tissue expression profiles to one another than singleton miRNAs and miRNAs in different families to one another (*P* = 9.6e–248 and 1.3e–200, Mann-Whitney *U* test). (**b**) The genes targeted by miRNAs within the same family are significantly more similar than among singleton and between family miRNAs (*P* ≈ 0 for both). (**c**) The diseases associated with human miRNAs in the same family are significantly more similar than between singleton miRNAs and miRNAs in different families (*P* ≈ 0 for both). (**d**-**f**) These results also hold when the miRNAs are stratified by age (*P* < 2e-16 for each, stratified Mann-Whitney *U* test)

To evaluate functional similarities within miRNA families compared to human singleton miRNAs, we computed the Jaccard similarity of the expression, gene target, and disease profiles of miRNAs in the same family and between singletons (Fig. 7a-c). miRNAs in the same family are significantly more similar in their tissues of expression, gene targets, and associated diseases than both miRNA singletons (*P* = 9.6e–248, *P* ≈ 0, and *P* ≈ 0, respectively, Mann-Whitney *U* test) and pairs of miRNAs in different families (*P* = 1.3e–200, *P* ≈ 0, and *P* ≈ 0).

**Fig. 8:**
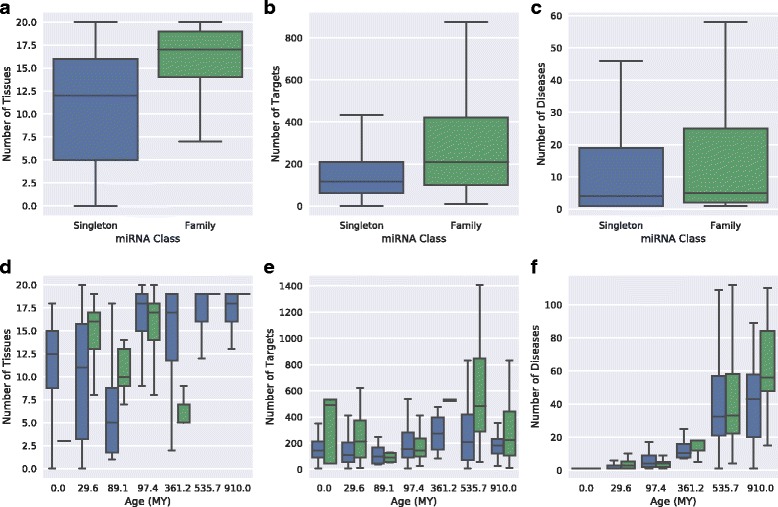
Comparison of the functional impact of human miRNAs in evolutionarily related families and singleton miRNAs. Human miRNAs in families of evolutionarily related miRNAs have a greater functional impact—as measured by breadth of expression, number of gene targets, and disease associations—than singleton miRNAs; however, this is largely due to their greater age. (**a**) Compared to human miRNA singletons, miRNAs in families of evolutionarily related miRNAs are expressed in more tissues (*P* = 1.8e–03; Mann-Whitney *U* test). (**b**) Human miRNAs in families target significantly more genes than singleton miRNAs (*P* = 3.8e–15). (**c**) Human miRNAs in families are associated with significantly more diseases than singleton miRNAs (*P* = 2.1e–28). (**d**-**f**) The differences between family and singleton miRNA are attenuated and less consistent when miRNAs are stratified by age (*P* = 0.067, 7.4e–05, and 1.5e–04, respectively; stratified Mann-Whitney *U* test). This suggests that differences in the age distribution of family vs. singleton miRNA likely contribute to their different functional impact

Furthermore, miRNAs in families, as a whole, are expressed in more tissues, feature more regulatory targets, and are implicated in more diseases than singletons (Fig. 8a-c; *P* = 1.8e–03, 3.8e–15, and 2.1e–28, respectively; Mann-Whitney *U* test)**.** This suggests a greater functional impact for miRNAs with multiple evolutionarily related human family members.

Several of our previous analyses demonstrated that the evolutionary age of a miRNA is often indicative of its functional attributes. Since family miRNAs are, as a group, older than miRNA singletons (Additional file 4), we evaluated whether the differences between family miRNAs and singletons hold when stratifying by age. Repeating the functional similarity analyses stratified by age did not change the results; miRNAs within the same family are consistently more functionally similar to each other in their tissue, gene target, and disease profiles than miRNA singletons are to one another and miRNAs in different families are to one another (Fig. 7e-f; *P* < 2e-16 for each, stratified Mann-Whitney *U* test). However, although our findings comparing family miRNAs to singletons without respect to age suggested that miRNAs within the same family are more functionally important, stratifying by age revealed that these differences are inconsistent and less significant within miRNA of the same age (Fig. 8d-f **;** *P* = 0.067, 7.4e–05, and 1.5e–04, stratified Mann-Whitney *U* test). This suggests that the greater functional impact of family vs. singleton miRNAs is partially driven by the fact that family miRNAs are older than singleton miRNAs.

In addition to comparing the number of gene targets for family and singleton miRNAs, we examined the functional roles of their gene targets. An average of 5% of gene targets associated with a family miRNA have transcription factor activity, compared to an average of 3.5% of singletons’ targets. This finding supports the greater functional influence of family miRNAs. It also suggests that miRNAs may target transcription factors less often than expected by chance.

### The genomic context and origins of old and young miRNAs

To explore whether genomic context mediates the functional roles of miRNAs of different ages, we intersected all miRNAs with human gene models. Overall, 38% of the miRNAs studied are located in introns. Old miRNAs are slightly less like to be located in introns than young miRNAs (33% vs. 40%). However, the difference is more pronounced when comparing family and singleton miRNAs; only 26% of family miRNAs are in introns, while 44% of singleton miRNAs are intronic. This suggests that there may be pressure against the formation and expansion of miRNA families in introns. Indeed, 33% of family miRNAs located in intronic regions are associated with disease, while only 15% of young miRNAs located in introns are disease-associated.

Precursor miRNAs (pre-miRNAs) are enriched for regulatory features, such as transcription factor binding sites (TFBS) [27]. We tested whether this enrichment varied by the age of the miRNAs. Old miRNAs are more likely to contain TFBS in their precursor sequences than young miRNAs. The average age of TFBS-associated miRNAs is 478.6 MY compared to an average of 169.9 MY across all miRNAs.

miRNAs are commonly created from sequences derived from transposable elements (TEs) [28]. Given that TEs make up a large fraction of the human genome and that TE-derived sequences are often young [29, 30], we hypothesized there might be differences in the age distribution of TE-derived and non-TE-derived miRNAs. To test this, we analyzed the association of miRNAs with annotated TEs in the human genome. Indeed, miRNAs that overlap annotated TE-derived sequences are significantly younger than non-TE-derived miRNAs (Additional file 5; average age 47.3 vs. 223.0 MY). Collectively, these results argue that old miRNAs have a different genomic distribution and functional characteristics than young miRNAs, and that this influences their propensity to cause disease.

### Evaluating trends in miRNA evolution and emergence across species is challenging due to lack of data

To evaluate if the patterns of miRNA emergence observed in human were conserved across other species and clades, we analyzed the ages for all annotated miRNAs from miRBase. We computed the ages of 12,952 miRNAs across 146 species. (We excluded 29 species available in miRBase due to a lack of TimeTree data.)

Over all miRNAs, the average age was 443.0 MY. While most miRNAs present in human were young—origins in the Homo, Hominidae, Catarrhini, and Boreoeutheria clades—the majority of all miRNAs present in miRBase were found to be older, originating on the branches leading from Boreoeutheria, Vertebrata, and Coelomata. This trend is likely due to the less comprehensive characterization of miRNAs in non-human species and the use of known human miRNAs to annotate other genomes. For instance, the number of miRNAs annotated in a species is significantly correlated with the percentage of young miRNAs (Spearman’s ρ = 0.68, *P* = 4.1e–06; see Additional file 6). This suggests that many clade-specific miRNAs are missing from the dataset. However, the peak in miRNAs originating in the Boreoeutheria clade in both the human and 146 species datasets suggests that miRNAs from this clade play key regulatory roles, consistent with previous hypotheses about the importance of miRNA in driving the evolution of vertebrate complexity [31, 32].

## Discussion

The phylogenetic age of a genetic element can provide insight into its functional significance. For example, older protein coding genes are more likely to be involved with disease than their younger counterparts [16, 17]. miRNAs evolve under significantly different functional and temporal dynamics than protein-coding genes; they turnover more rapidly and are significantly younger than genes [18, 19]. Nonetheless, we demonstrated that, as for genes, there is a strong correlation between miRNA age and disease association. The greater number of diseases associated with older miRNAs compared to younger miRNAs argues that they are more likely to regulate critical functional pathways. (However, many young miRNAs also contribute to disease [7].) Evaluation of miRNAs associated with individual diseases reinforced the similarity of the evolutionary histories of miRNAs associated with disease and the histories of disease genes. For example, many diseases, such as cancers, that have been associated with primarily older genes [24], also are influenced by older miRNAs.

Consistent with the greater association of older miRNA with disease, we found that older miRNAs are generally expressed in more tissues than young miRNAs, and older miRNAs are also associated with more gene targets. Older miRNAs are also generally broadly expressed, while in contrast, younger miRNAs exhibit variation in the number of tissues in which they are expressed, with some broadly expressed and others tissue-specific (Fig. 6). The enrichment for TFBS sites in the pre-miRNA sequence of older miRNAs compared to younger miRNAs is also suggestive of their robust regulatory role and may support a feedback mechanism that enables their functions in developmental programming [33, 34]. These results argue that old miRNAs generally have greater functional impact than young miRNAs.

Our findings that the breadth of a miRNA’s expression across tissues and its number of gene targets are correlated with age are consistent with several recent reports and a model in which miRNAs increase in breadth of expression as they age [21, 22, 35]. However, it is also possible that broadly expressed miRNAs are more likely to be maintained over time due to their larger regulatory domains. Indeed, other studies have argued for increasing refinement of miRNA targeting and function with age [5, 22]. These differences are due, at least in part, to our incomplete knowledge of miRNAs and different methods for identifying targets. For example, many of these investigations into miRNA targeting and its effects on cellular function have relied on computational predictions of miRNA-target pairs. Unfortunately, target prediction algorithms have not achieved sufficient accuracy and consistency to enable solid conclusions [3, 21]. Thus, we analyzed only experimentally validated targets, which are certainly incomplete. It is also possible that computational and experimental target prediction methods have different biases with respect to functionally relevant targets vs. unconstrained target interactions. As such, stronger curation efforts and algorithm development are needed to consolidate the breadth of available miRNA data and enable replicable and consistent studies of the mechanics of miRNA-target acquisition. In aggregate, we argue that human miRNAs gain functionally relevant targets as they age—usually older genes—but many additional factors, such as the tissues of expression and the presence of evolutionarily related family members, influence the diversity of their functional interactions.

Evolutionarily related human miRNAs are more similar to one another in their tissue expression profiles, gene targets, and disease associations than the similarity of pairs of singleton miRNAs and miRNAs in different families, regardless of age. These results are relevant to recent studies of the functions and evolutionary dynamics of physically clustered miRNA [10, 15, 26], since many of the constituent members of clusters are created via duplication. For example, a simple comparison of family and singleton miRNAs that ignored their age suggested that miRNAs from families have a greater functional influence; however, stratifying miRNAs by age revealed that these trends are not consistent over evolutionary time (Fig. 8) and that the apparent difference was due to the older average age of family miRNAs.

The genomic context of a miRNA is linked to its evolutionary history and can be informative about its functional significance. For example, intronic miRNAs may serve as negative feedback regulators of their constituent genes [36, 37]. This association is particularly relevant when considering family miRNAs, for which negative regulatory effects might be amplified due to similar seed sequences. This possibility is supported by our findings; fewer family miRNAs are located in introns compared to singletons, and family miRNAs in introns have a greater propensity to be associated with disease.

Furthermore, there is also growing evidence that TEs have played a key role in miRNA biogenesis and the evolution of gene regulation [28, 30, 34]. For example, the majority of primate-specific regulatory sequences as mapped by DNase I hypersensitivity overlap sequences derived from TEs [29]. Our results support a similar conclusion for miRNAs; TE-derived miRNAs are significantly younger than non-TE miRNAs (Additional file 5).

Expanding analysis of miRNA evolutionary and functional dynamics to non-model species will be challenging due to our incomplete knowledge of the miRNAs present in most species. There are 1025 human miRNAs annotated in the miRBase dataset analyzed here, but the average number of miRNAs annotated per species is 88.7. Analyses of miRNAs derived from RNA-seq data from several tissues across different mammals revealed an expansion of the miRNA complement in mammals [18], but this is unlikely the driver of the large difference in miRNA number. Indeed, we identified a strong correlation between the number of miRNAs with origins in the lower quartile of the possible age range and the total number of miRNAs annotated for a given species (Additional file 6; Spearman’s ρ = 0.68, *P* = 4.1e–06). The differences in the age distributions of miRNAs across species observed in our study highlight the need for more comprehensive identification of the miRNAs encoded in different genomes; this will be critical for future evolutionary analyses of miRNA origins and function.

## Conclusions

Our analyses illustrate the importance of considering miRNA origins and genomic context in comparisons of miRNAs and their functions. miRNAs retain considerable untapped potential as disease markers and avenues for therapeutic intervention in combatting human disease, and we believe that integrating analysis of their evolutionary histories with traditional molecular and cellular characterizations will help achieve this goal.

## Methods

To compute evolutionary ages for miRNAs and explore their known associations with disease in humans, we integrated several datasets and computational tools. In outline, first, we identified families of evolutionarily related miRNAs. Next, we determined the phylogenetic age of miRNAs based on their distribution across species. Finally, we obtained miRNA-disease, miRNA-target, and miRNA-tissue expression data and examined these relationships in the evolutionary context.

### miRNA families and phylogenetic trees

Data for all miRNAs were downloaded from miRBase v21, an online database of annotations and sequences for published miRNAs across many species [1, 38]. We extracted all species for which miRNA were annotated by miRBase and used the phyloT web server to generate a Newick format phylogenetic tree for 175 species with miRNA data [39]. Annotation of miRNAs to evolutionarily related families also came from miRBase. For family-based analyses, we report results for miRNA families with more than four members, since many families with fewer members lacked functional data; however, including all families did not influence our main conclusions.

### Assigning evolutionary ages to human miRNAs

ProteinHistorian (PH) is a web server and command line tool we previously developed to determine the evolutionary ages for eukaryotic proteins and to test for enrichment of specific evolutionary origins in protein sets of interest [23]. Using families of homologous proteins and a phylogenetic tree that captures the evolutionary relationships of the species under consideration, PH provides several algorithms for estimating the ancestral branch on which each protein emerged based on its presence or absence across species. To calculate miRNA ages, we adapted the PH approach to analyze groups of evolutionarily related miRNAs across species obtained from mirBase. Although PH was initially designed to determine eukaryotic protein ages, its modular design made it straightforward to adapt to this new setting. We employed the Dollo parsimony approach in our adapted PH to estimate miRNA ages based on growing evidence from the literature that human miRNAs are only rarely lost once permanently added to a lineage’s genome [40, 41]. Applying this approach to 286,645 miRNAs across all species considered from miRBase, we estimated the branch of the phylogenetic tree on which each first appeared. To calibrate the timing of each of these evolutionary branches, we used expert estimates from TimeTree for the divergence of different species [42]. When expert estimates were not available from the TimeTree, the average of published estimates was used. We refer to miRNAs less than 100 million years old as “young” and those over 100 million years old as “old” or “ancient”. We compared the miRNA ages to the ages of human genes calculated by PH using the asymmetric Wagner parsimony criteria over alignments of proteins from 48 species [23].

### Analyzing the relationship between miRNA age and disease association

We downloaded miRNA-disease associations from the Human microRNA Disease Database version 2 (HMDD), last updated in June of 2014 [8]. HMDD associated 578 human miRNAs with 383 diseases. We evaluated the correlation of the age of each miRNA annotated in miRBase with the number of its associated diseases in the HMDD with Spearman’s rank correlation. Since the HMDD disease set contained many similar diseases, we also computed a “reduced” disease set for each miRNA in which cancers were merged. The reduced set contained 302 entries. We also mapped diseases to their 2017 medical subject headings (MeSH) vocabulary from NCBI, and the associated hierarchy was used to collapse diseases at different levels of specificity. Disease names and mappings are given in Additional file 3.

In order to evaluate the similarity of the diseases associated with different miRNA families, we associated each miRNA with a binary vector indexed by disease in which a “1” indicated association with the disease, and “0” indicated no association. We then computed the Jaccard similarity index between the binary vectors of miRNAs within the same age class. This method was extended when comparing the disease profiles of miRNA families and miRNA singletons. For all age-stratified analyses comparing family and singleton miRNAs, we used the stratified Mann-Whitney U test as implemented in the sanon package in the R programming language [43].

### miRNA targets and their evolutionary histories

We downloaded 319,690 experimentally-verified miRNA target interactions from miRTarBase version 21 in May 2016 [25]. In order to evaluate how miRNAs related to their targets in terms of evolutionary history, we downloaded previously computed evolutionary ages for the targets from the PH database [23]. We identified human genes with transcription factor activity using annotations from the Gene Ontology Consortium downloaded on January 7, 2017 [44]. To evaluate whether relationships between the age of miRNA and their target genes were simply a consequence of the differences in the age distributions of miRNAs and genes, we performed a permutation analysis by randomly shuffling the assignments between miRNAs and targets 10,000 times. A binary Jaccard similarity approach similar to the one described above was also employed in evaluating relationships between miRNAs and their targets.

### Expression patterns of miRNAs in different tissues

We downloaded miRNA expression data in 20 tissues from miRmine in May 2016 [45]. Since the miRmine database featured multiple profiles for each tissue presented, we consolidated the expression data within each tissue to produce a summary for each miRNA indicating the presence or absence of a miRNA in a given tissue. The tissue expression profiles were compared with the Jaccard similarity index.

### Genomic attributes of miRNAs stratified by age and disease class

Intron and exon boundaries were taken from the knownCanonical track of the UCSC Genes human gene set from the UCSC Genome Browser [46]. Evolutionary sequence conservation was evaluated using the PhastCons elements computed over a multiple sequence alignment of 99 vertebrate whole genome sequences with the human genome (phastConsElements100way) [47]. We downloaded transcription factor binding sites in miRNA precursor sequences and TE associations with annotated miRNAs from previous studies [27, 48].

## Additional files

Additional file 1: miRBase Species List. List of all species used in our phylogenetic analysis, along with their respective miRBase codenames and number of annotated miRNAs. (XLSX 35 kb)

Additional file 2: Human miRNA Ages. The ages of all human miRNAs computed by ProteinHistorian, with timing estimates in millions of years (MY) based on calibrations from TimeTree. (TXT 18 kb)

Additional file 3: miRNA Disease Associations. List of diseases compiled from HMDD, the number of human miRNAs associated with each disease, and the corresponding MeSH term for each disease. The file also includes average and median ages for disease miRNAs and Mann-Whitney *U* statistics comparing the ages of the miRNAs associated with each disease to the background set of all miRNAs. The “Collapsed” column indicates if the disease was collapsed into a single disease in the cancer-control analysis. (XLSX 33 kb)

Additional file 4: The phylogenetic age distribution of family miRNAs versus singletons. The median age of all family miRNAs (89.1 MY) is significantly older than the median age of singletons (29.6 MY; *P* = 4.6e–5, Mann-Whitney *U* test). (PDF 21 kb)

Additional file 5: miRNAs derived from transposable elements (TEs) are significantly younger than non-TE-derived miRNAs. TE-derived miRNAs have an average age of 47.3 MY, while the non-TE-derived miRNAs have an average age of 223.0 MY. (PDF 13 kb)

Additional file 6: The number of miRNAs annotated in a species is significantly correlated with the percentage of young miRNAs in the species. Over 146 species, we observed a Spearman’s correlation of 0.68 (*P* = 4.1e–06) between the number of annotated miRNAs and the percentage of young miRNAs in the species. This relationship is likely the result of limitations in current knowledge of miRNA sequences and annotations. In this analysis, young miRNAs were defined as those younger than 25 % of the overall age range. See Additional file 1 for the list of all species considered and miRNA counts. (PDF 15 kb)

## Abbreviations

HMDD: Human microRNA Disease Database
miRNA: microRNA
PH: ProteinHistorian
TE: Transposable element
TFBS: Transcription Factor Binding Site
