# Supplementary figures and images for: Ancient human miRNAs are more likely to have broad functions and disease associations than young miRNAs
Source: BMC Genomics. 2017 Aug 31;18:672. doi: 10.1186/s12864-017-4073-z (PMC5579935; doi:10.1186/s12864-017-4073-z)

# Protein Age Distribution Comparison

Mann-Whitney U test:  $U = 9.8e+04$  ( $p = 4.55e-05$ )

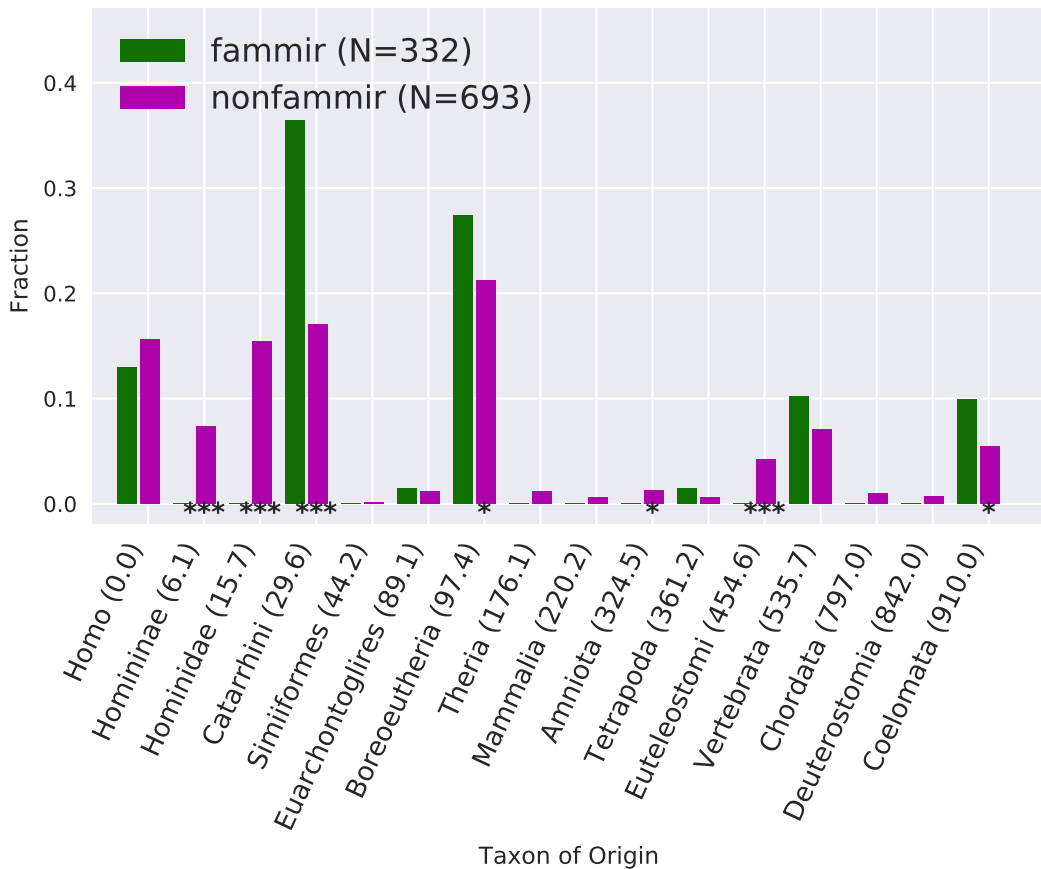

Supplement: Supplementary file 4 — The phylogenetic age distribution of family miRNAs versus singletons. The median age of all family miRNAs (89.1 MY) is significantly older than the median age of singletons (29.6 MY; P = 4.6e–5, Mann-Whitney U test). (PDF 21 kb) [file 12864_2017_4073_MOESM4_ESM.pdf]

Age Differences Between TE-Derived and Other miRNAs

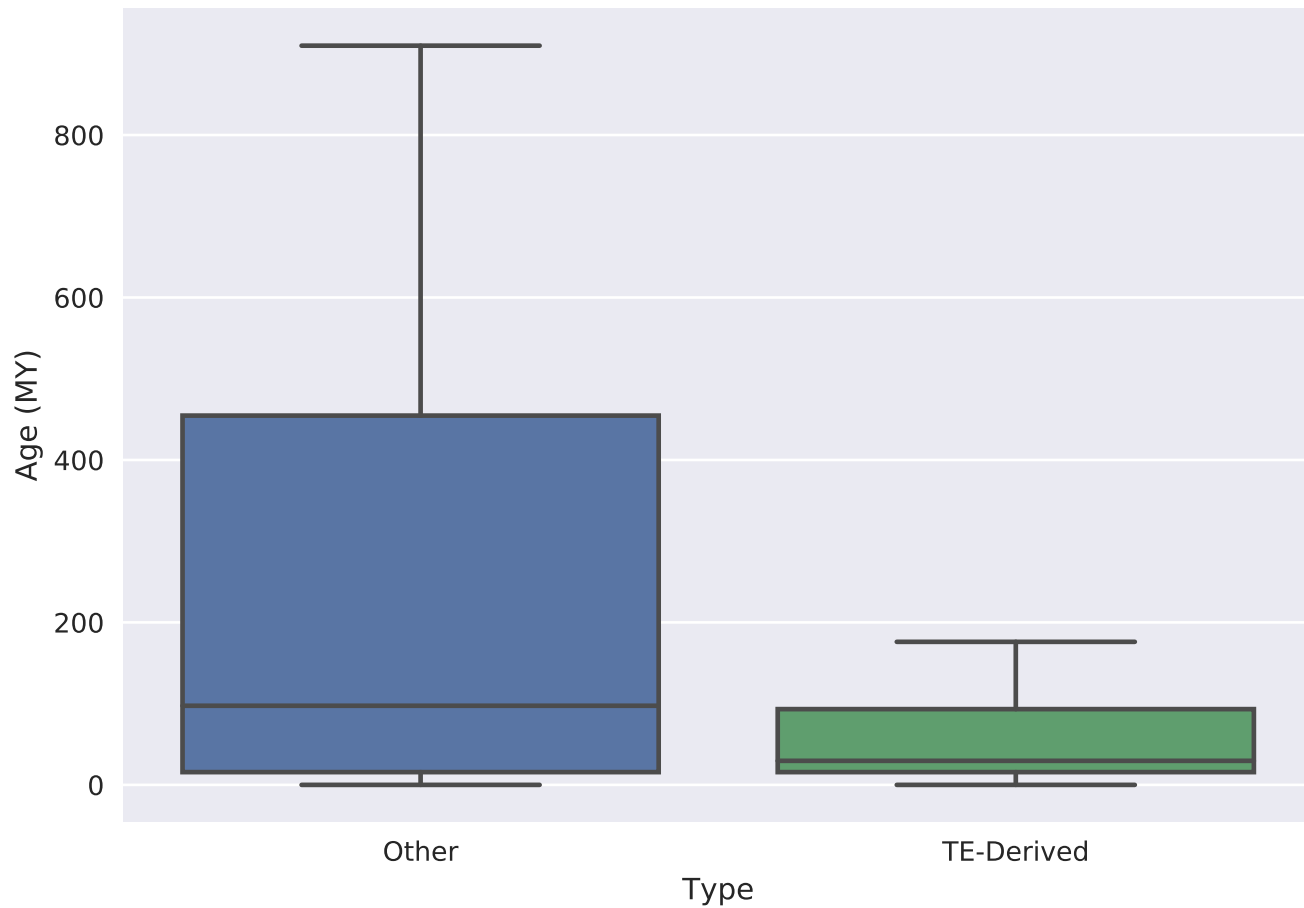

Supplement: Supplementary file 5 — miRNAs derived from transposable elements (TEs) are significantly younger than non-TE-derived miRNAs. TE-derived miRNAs have an average age of 47.3 MY, while the non-TE-derived miRNAs have an average age of 223.0 MY. (PDF 13 kb) [file 12864_2017_4073_MOESM5_ESM.pdf]

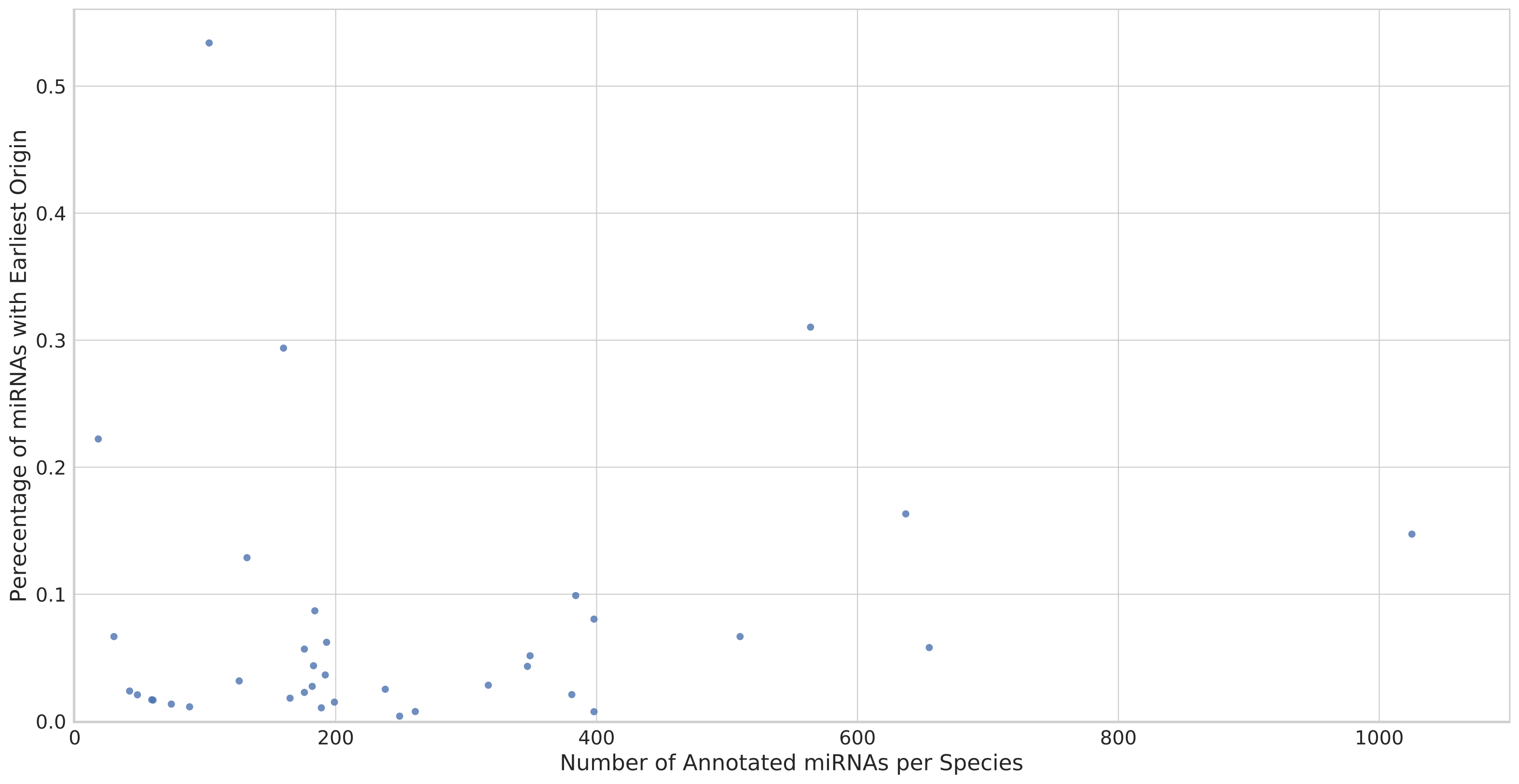

Supplement: Supplementary file 6 — The number of miRNAs annotated in a species is significantly correlated with the percentage of young miRNAs in the species. Over 146 species, we observed a Spearman’s correlation of 0.68 (P = 4.1e–06) between the number of annotated miRNAs and the percentage of young miRNAs in the species. This relationship is likely the result of limitations in current knowledge of miRNA sequences and annotations. In this analysis, young miRNAs were defined as those younger than 25 % of the overall age range. See Additional file 1 for the list of all species considered and miRNA counts. (PDF 15 kb) [file 12864_2017_4073_MOESM6_ESM.pdf]
